# Immunotherapy for Alzheimer’s disease: targeting β-amyloid and beyond

**DOI:** 10.1186/s40035-022-00292-3

**Published:** 2022-03-18

**Authors:** Chenghuan Song, Jiyun Shi, Pingao Zhang, Yongfang Zhang, Jianrong Xu, Lanxue Zhao, Rui Zhang, Hao Wang, Hongzhuan Chen

**Affiliations:** 1 Department of Pharmacology and Chemical Biology, Shanghai Jiao Tong University School of Medicine, Shanghai, 200025 China; 2 Shanghai Universities Collaborative Innovation Center for Translational Medicine, Shanghai Jiao Tong University School of Medicine, Shanghai, 200025 China; 3 Key Laboratory of Cell Differentiation and Apoptosis of Chinese Ministry of Education, Shanghai Jiao Tong University School of Medicine, Shanghai, 200025 China; 4 Academy of Integrative Medicine, Shanghai University of Traditional Chinese Medicine, Shanghai, 201203 China; 5 Department of Clinical Pharmacy, Institute of Interdisciplinary Integrative Medicine Research, Shuguang Hospital, Shanghai University of Traditional Chinese Medicine, Shanghai, 201203 China

**Keywords:** Alzheimer’s disease, Immunotherapy, Vaccine, Antibody, Drug development

## Abstract

Alzheimer’s disease (AD) is the most common neurodegenerative disease in the elderly worldwide. However, the complexity of AD pathogenesis leads to discrepancies in the understanding of this disease, and may be the main reason for the failure of AD drug development. Fortunately, many ongoing preclinical and clinical studies will continually open up avenues to unravel disease mechanisms and guide strategies for AD diagnosis and drug development. For example, immunotherapeutic strategies targeting amyloid-β (Aβ) and tau proteins were once deemed almost certainly effective in clinical treatment due to the excellent preclinical results. However, the repeated failures of clinical trials on vaccines and humanized anti-Aβ and anti-tau monoclonal antibodies have resulted in doubts on this strategy. Recently, a new anti-Aβ monoclonal antibody (Aducanumab) has been approved by the US Food and Drug Administration, which brings us back to the realization that immunotherapy strategies targeting Aβ may be still promising. Meanwhile, immunotherapies based on other targets such as tau, microglia and gut-brain axis are also under development. Further research is still needed to clarify the forms and epitopes of targeted proteins to improve the accuracy and effectiveness of immunotherapeutic drugs. In this review, we focus on the immunotherapies based on Aβ, tau and microglia and their mechanisms of action in AD. In addition, we present up-to-date advances and future perspectives on immunotherapeutic strategies for AD.

## Introduction

Alzheimer’s disease (AD), the most prevalent cause of dementia in the elderly, is pathologically characterized by extracellular amyloid-β (Aβ) plaques, hyperphosphorylated tau in neurofibrillary tangles and neuroinflammation [1, 2]. The clinical symptoms of AD patients mainly include cognitive dysfunction and memory loss [3]. Most AD cases have an onset after 65 years of age, accounting for 5%–10% of this age population, and this number increases to 50% in people older than 85 years [4]. AD patients suffer progressive disability due to dementia and movement disorders caused by AD, and eventually die within 5–12 years after onset [5]. According to the current theories, AD pathologies are driven by both modifiable and non-modifiable risk factors. The modifiable risk factors include, but are not limited to, disorders such as diabetes, hypertension and cardiovascular diseases, and an unhealthy lifestyle also raises the risk of disease. Aging and genetic factors, such as the apolipoprotein E (*APOE*) gene, are the main non-modifiable risk factors for AD [6–9].

Current commonly used drugs for AD, mainly cholinesterase inhibitors and N-methyl-*D*-aspartate receptor antagonists, are symptomatic and still not effective in curbing the disease progression [10, 11]. Much effort is needed to develop therapeutic methods for AD. The disease-modifying treatments have attracted much attention. According to the Aβ cascade hypothesis, some researchers believe that active immunization with vaccines or passive immunization with specific antibodies that aims to promote Aβ clearance is promising, while others hold the opinion that targets beyond Aβ, such as tau, are imperative. In addition, some microglia-based immunotherapies targeting various immunological molecules such as triggering receptor expressed on myeloid cells-2 (TREM2), CD38 and Toll-like receptors (TLRs) are under investigation as well [12–15].

Both active and passive immunization strategies have advantages. Active immunotherapy (vaccines) depends on the cellular and humoral immune responses, resulting in long-term generation of endogenous antibodies. Compared with passive immunotherapy, active immunotherapy produces high-concentration antibodies in the human body, with few injection times and less medical cost. On the other hand, passive immunotherapy (antibodies) is regarded more suitable and effective for elderly patients whose responsiveness to vaccines is reduced. Furthermore, upon occurrence of adverse reactions, the effects of humanized monoclonal antibodies can be stopped more conveniently than vaccines due to the targeting of specific protein conformations. However, passive immunization requires repeated dosing and more expenditure. In addition, both approaches may induce over-activation of the innate and adaptive immune systems, resulting in side effects such as cerebral vasculitis [16]. In this review, we introduce immunotherapeutic strategies that have already been approved or under clinical trials, discuss their mechanisms of action, and propose the perspectives and challenges of immunotherapies for AD.

## Immunotherapies based on Aβ

Aβ has been thought to play a pivotal role in AD pathogenesis. It causes synaptic impairment and neurodegeneration, consequently contributing to the cognitive dysfunction observed in AD [17–19]. Therefore, strategies targeting Aβ could effectively curb the progression of AD. At present, the mechanisms of action of anti-Aβ drugs mainly include reducing Aβ production, preventing Aβ aggregation and promoting Aβ clearance.

During the past years, many agents have been investigated for their ability to decrease Aβ production and inhibit Aβ agglomeration. However, all have failed, leading to the reconsideration of whether Aβ is still a critical therapeutic target and worthy of further studies. Considering that Aβ accumulation is the main cause of the neurodegenerative process in AD, accelerating its clearance may crawl on the right way. Therefore, immunotherapy has become the focus of exploration to promote Aβ clearance and has greatly inspired research on anti-Aβ therapies [20–24].

**Fig. 1:**
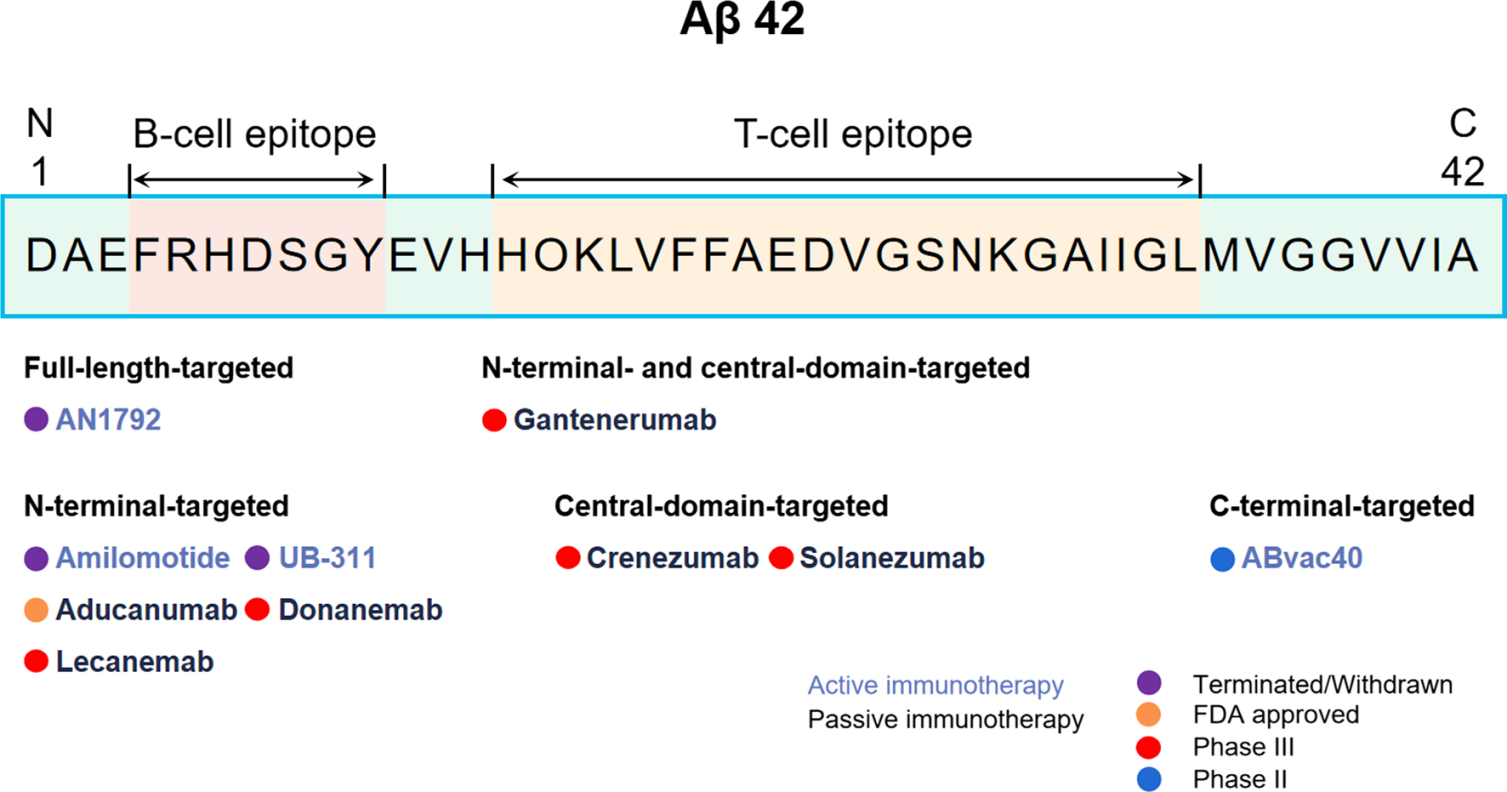
Immunotherapy strategies targeting Aβ. Aβ immunotherapies are classified by the mechanism of action, Aβ domain targets and progress of development

Currently, the most elaborated anti-Aβ immunotherapies are vaccines and exogenous antibodies, known as active and passive immunotherapy, respectively. Active immunization stimulates the immune system by administering Aβ or its fragments, thereby triggering an immune response to produce endogenous antibodies against Aβ [25]. In 1999, active immunization against full-length Aβ was first reported to be effective in reducing Aβ deposition in the brains of PDAPP mice [26]. After that, AN1792, a vaccine targeting full-length Aβ, was developed and entered clinical trials. However, the trials were terminated due to the occurrence of T cell-mediated meningoencephalitis in 6% of the recruited patients with moderate-to-severe AD [27, 28]. To reduce an overactive immune reaction, the second generation of vaccines without T-lymphocyte epitope was developed, such as CAD106. The active immunotherapy has an advantage of short-term drug administration with long-lasting antibodies at a limited cost, but at the same time, the immune responses and adverse reactions are hard to predict, especially in the elderly. Some current progress of the development of active immunotherapy for AD is shown in Fig. 1 and Table 1.

**Table 1:** Immunotherapies targeting Aβ in AD drug development

Therapeutic strategy	Drug	Mechanism	Sponsor	Study population	Admin	Phase	Results	Clinical Trial Identifier	Start date	Estimated end date
Active immunotherapy	AN1792	Vaccination	Janssen/Pfizer	Mild to moderate AD	IM	II	Terminated	NCT00021723	2001 Sept	2003 Sept
	Amilomotide (CAD106)	Vaccination	Novartis	Participants at risk of the onset of clinical symptoms of AD	IM	II/III	Terminated	NCT02565511	2015 Nov	2020 Apr
	UB-311	Vaccination	United Neuro-science	Mild AD	IM	II	Completed	NCT02551809	2015 Oct	2018 Aug
				Mild AD		II	Terminated	NCT03531710	2018 Aug	2019 Oct
	ABvac40	Vaccination	Araclon Biotech	Participants with amnestic mild cognitive impairment or very mild AD	SC	II	Active, not recruiting	NCT03461276	2018 Feb	2022 Dec
Passive immunotherapy	Solanezumab (LY2062430)	Monoclonal antibody	Eli Lilly	Mild to moderate AD	IV	III	Completed	NCT00905372	2009 May	2012 Apr
				Mild to moderate AD			Completed	NCT00904683	2009 May	2012 Jun
				Mild to moderate AD			Terminated	NCT01127633	2010 Dec	2017 Feb
				Mild AD		III	Terminated	NCT01900665	2013 Jul	2016 Oct
				Prodromal AD		III	Terminated	NCT02760602	2016 Jun	2017 May
				Participants at risk of memory loss		III	Active, not recruiting	NCT02008357	2014 Feb	2022 Dec
	Gantenerumab	Monoclonal antibody	Roche	Prodromal AD	IV		Completed	NCT01224106	2010 Nov	2020 Sept
				Mild AD			Completed	NCT02051608	2014 Mar	2021 Apr
				Prodromal to mild AD			Recruiting	NCT04374253	2014 Mar	2021 Apr
				Early AD			Recruiting	NCT03444870	2018 Jun	2023 Nov
				Early AD			Active, not recruiting	NCT03443973	2018 Aug	2022 Sept
				Early AD			Active, not recruiting	NCT04339413	2020 May	2023 Apr
	Aducanumab (BIIB037)	Monoclonal antibody	Biogen	Early AD	IV	III	Terminated	NCT02484547	2015 Sept	2019 Aug
				Early AD		III	Terminated	NCT02477800	2015 Aug	2019 Aug
				Early AD		III	Active, not recruiting	NCT04241068	2020 Mar	2023 Oct
	Crenezumab (RG7412)	Monoclonal antibody	Roche/AC Immune SA	Prodromal to mild AD	IV	III	Terminated	NCT02670083	2016 Mar	2019 May
				Prodromal to mild AD		III	Terminated	NCT03114657	2017 Mar	2019 Jun
				Prodromal to mild AD		III	Terminated	NCT03491150	2018 Apr	2019 May
	Lecanemab (BAN2401)	Monoclonal antibody	Biogen /Eisai	Early AD	IV	III	Recruiting	NCT03887455	2019 Mar	2024 Aug
				Preclinical AD		III	Recruiting	NCT04468659	2020 Jul	2027 Oct
	Donanemab (LY3002813)	Monoclonal antibody	Eli Lilly	Early symptomatic AD	IV	III	Recruiting	NCT04437511	2020 Jun	2023 Dec
				Preclinical AD		III	Recruiting	NCT05026866	2021 Aug	2027 Sept

AD, Alzheimer’s disease; Admin, Route of administration; SC, subcutaneous; IM, intramuscular; IV, intravenous

Due to the low reactivity of vaccines and the emergence of T cell-dependent adverse reactions, much effort has been put to passive immunotherapy using humanized monoclonal antibodies or polyclonal immunoglobulins to promote Aβ clearance [29]. The passive immunotherapy ensures relatively consistent antibody titers, but is usually accompanied by vasogenic oedema, cerebral amyloid angiopathy with microhemorrhages, and other adverse reactions. The generally considered mechanisms of passive immunization include antibody opsonization of the antigen, which causes macrophage phagocytosis and complement activation; antibody-mediated peripheral reduction of Aβ in favor of Aβ efflux from the central nervous system; antibody-catalyzed modification of the secondary structure of Aβ monomers to block the formation of oligomers or fibrils; and Fc receptor-medicated outflow of antigen–antibody complexes across the blood–brain barrier [29–33].

The first Aβ antibody was tested *in vivo* in 2000 by Bard F et al., and the results showed that peripheral administration of the antibody against the N-terminus of Aβ was able to induce microglia-mediated Aβ phagocytosis, thus ameliorating the Aβ-related pathology in an AD mouse model [34]. After that, Aβ antibodies targeting the N-terminus and other regions, such as mid-region and C-terminus, have been tested for their ability to promote Aβ clearance and prevent cognitive dysfunction in animal models. Nowadays, antibodies specific for a single small peptide sequence of Aβ, have shown some therapeutic effects. In addition to aducanumab that has just been approved for marketing, six monoclonal antibodies sponsored by Biogen, Eli Lilly, Eisai, and Roche have entered phase III trials. In addition, two vaccines from Araclon Biotech and AC Immune SA, respectively, have reached phase II trials (Fig. 1 and Table 1). Monoclonal antibodies LY3372993 from Eli Lilly, RO7126209 from Roche, and SHR-1707 from Jiangsu Hengrui Pharmaceuticals are under investigation in phase I trials.

### Active immunotherapy

#### AN1792

AN1792 was the first anti-Aβ vaccine tested clinically. It is a synthetic full-length Aβ_42_ with QS-21 adjuvant. In a phase IIa clinical trial (NCT00021723), 19.7% of AN1792-treated patients were antibody responders with high anti-AN1792 IgG titers. However, although AN1792 treatment reduced Aβ deposition and showed positive effects in neuropsychological test battery (NTB) score and cerebrospinal fluid (CSF) tau levels, this difference was only found in the antibody responders. Moreover, AN1792 injection resulted in T cell-mediated meningoencephalitis in 6% of treated participants, which led to the termination of this clinical study [35, 36]. After that, another long-term follow-up study was conducted and found that the antibody responders defined in the previous phase IIa clinical trial maintained low but detectable, sustained anti-AN1792 antibody titers, which contributed to the less functional decline and long-term functional benefits [37].

#### Amilomotide

Amilomotide (CAD106) is a vaccine that comprises N-terminal Aβ_1-6_ as a B-cell epitope to generate anti-Aβ antibodies without an Aβ-specific T-cell response [38]. The phase I trial (NCT00411580) found that CAD106 had a favorable safety profile and an acceptable antibody response [38]. Phase II trials, including IIa (NCT00733863, NCT00795418, NCT00956410, NCT01023685) and IIb (NCT01097096), suggested an appropriate balance between antibody response and tolerability. However, as the first vaccine entering phase II/III trial (NCT02565511), CAD106 caused unpredicted changes in cognitive function, brain volume, and body weight, leading to early study termination.

#### UB-311

UB-311 comprises two synthetic Aβ_1-14_-targeting peptides as B-cell epitopes, each conjugated to different helper T-cell peptide epitopes (UBITh®). The Th2-biased delivery system is applied to maximize immunogenicity and minimize T-cell inflammatory reactivity [39]. Results of a phase II trial (NCT02551809) indicated that UB-311 had the potential to improve cognitive function in early-to-mild AD patients with 100% responder rate and strong on-target immunogenicity [39]. However, this clinical trial did not include a placebo group. Rather, it compared the increase of Alzheimer’s Disease Assessment Scale-Cognitive Section (ADAS-Cog) scores from baseline in the subgroup of mild AD patients (Mini-Mental State Examination [MMSE] score ≥ 20) with the moderate AD subgroup. Another phase II trial (NCT03531710) was terminated due to treatment assignment error.

### Passive immunotherapy

#### Aducanumab

Aducanumab (BIIB037) is a human IgG1 monoclonal antibody that binds to the N terminus of Aβ in an extended conformation [40]. It targets Aβ aggregates, including soluble oligomers and insoluble fibrils. The phase Ib randomized trial, PRIME (NCT01677572), showed significant reductions in amyloid positron emission tomography (PET) standard uptake value ratio (SUVRr) composite score in the aducanumab-treated patients, especially in those treated with 10 mg/kg aducanumab at 54 weeks. The brain amyloid burden decreased in a dose- and time-dependent manner in patients with prodromal or mild AD. The Clinical Dementia Rating-Sum of Boxes (CDR-SB) and MMSE scores were delayed by aducanumab treatment, indicating a positive effect on cognition and clinical progression [41].

Aducanumab achieved convincing results in PRIME, although the amyloid-related imaging abnormalities vasogenic edema (ARIA-E) occurred dose-dependently in 3%–41% of aducanumab recipients and was more common in APOE ε4 carriers [41]. The phase II study was skipped owing to the promising phase I data. Two identically designed phase III studies ENGAGE (NCT02477800) and EMERGE (NCT02484547) were conducted but both were terminated in March 2019 based on futility analysis indicating little likelihood of treatment efficacy [42]. However, a reversal occurred in October 2019. An expanded analysis revealed that EMERGE met its primary endpoint, where patients in the high-dose group showed a statistically significant reduction of clinical decline from baseline in CDR-SB scores by 22% at 78 weeks. Although ENGAGE did not achieve its primary endpoint, data from patients receiving high-dose aducanumab were consistent with the findings of EMERGE [43]. It is controversial whether the positive results of EMERGE are sufficient to establish validity in the context of the negative results of ENGAGE.

In March 2021, three members of the US Food and Drug Administration (FDA) Peripheral and Central Nervous System Drugs Advisory Committee published their opposition to aducanumab for AD treatment in a *JAMA* article [44]. However, aducanumab was still given official approval to treat AD based on the surrogate endpoint, i.e., removal of amyloid plaques from the brain, by the U.S. FDA through the accelerated approval pathway in June 2021. Although according to the accelerated approval provisions, a new phase III clinical trial (NCT04241068) is still needed to evaluate the efficacy and safety, aducanumab is the first new anti-AD drug to win FDA approval since memantine launched in 2003. After the approval, many researchers and drug developers considered the FDA’s decision on aducanumab is wrong and will ultimately undermine confidence in the agency. Anyway, aducanumab is now the first available therapy to target and ameliorate the fundamental disease process of AD. Meanwhile, the approval of aducanumab has brought new hope to patients in the early stage of AD and shed new light on the research and innovation to conquer the disease.

#### Donanemab

Donanemab (LY3002813) is a humanized monoclonal IgG1 antibody that binds specifically to the N-terminal pyroglutamate Aβ epitope, which is present merely in deposited Aβ. In the phase II trial, TRAILBLAZER-ALZ (NCT03367403), donanemab induced a smaller reduction of integrated Alzheimer’s disease rating scale score in patients with early-stage AD, signifying less cognitive and functional decline, although results for secondary outcomes were mixed. Moreover, results of ^18^F-florbetapir PET showed that patients treated with donanemab displayed a significant reduction of amyloid plaque at 76 weeks and 54.7% of the participants had an amyloid-negative status at 52 weeks [45]. The greater amyloid plaque reduction driven by donanemab treatment was highly associated with less cognitive decline and decreased tau progression at 24 weeks. Meanwhile, within 12 weeks of donanemab treatment, a rapid decline in plasma P-tau217, a biomarker for AD pathology, was observed. Due to the higher incidence of ARIA-E in the donanemab group than in the placebo group (26.7% *vs* 0.8%), larger and longer trials are needed to evaluate the efficacy and safety of donanemab in AD. Currently, a follow-on study, TRAILBLAZER-EXT (NCT04640077), is underway for patients enrolled in TRAILBLAZER-ALZ. And a phase III study, TRAILBLAZER-ALZ 2 (NCT04437511) with 1500 participants, is being conducted to specifically assess whether donanemab can prevent the clinical progression of patients with pathological evidence of AD but yet to show clinical symptoms. Another phase III study, TRAILBLAZER-ALZ 3 (NCT05026866) with 3300 participants with preclinical AD, has been started to further determine the safety and efficacy of donanemab.

#### Lecanemab

Lecanemab (BAN2401) is a humanized IgG1 monoclonal antibody preferentially targeting soluble aggregated Aβ and possessing activity across oligomers, protofibrils, and insoluble fibrils. In the phase II trial, BAN2401-G000-201 (NCT01767311), although the 12-month primary endpoint was not met, the brain amyloid plaques were reduced, and several clinical and biomarker endpoints showed sustained clinical remission at the highest dose of 10 mg/kg biweekly. The reduction of amyloid PET SUVr value and clinical decline on Alzheimer’s Disease Composite Score (ADCOMS) and ADAS-Cog14 were dose-dependent over 18 months. A confusing finding was that the difference in CDR-SB decline between the lecanemab and placebo groups was not significant at 18 months, but significant at 12 months. According to the Bayesian sensitivity analyses, in comparison to the placebo, lecanemab at a biweekly dose of 10 mg/kg induced greater reductions of cognitive decline in APOE4 carriers *versus* non-carriers. CSF biomarker analyses showed an increase in Aβ_42_ and a decrease in p-tau compared with placebo, but results on total tau were inconsistent between 12 and 18 months. It is worth noting that the ARIA-E incidence was 9.9% at 10 mg/kg in the overall population and was 14.3% in APOE4 carriers, indicating well tolerance of lecanemab [46]. A phase III study, Clarity AD (NCT03887455), is underway to evaluate the efficacy, long-term safety, and tolerability of lecanemab in early AD. Another phase III trial, AHEAD 3–45 (NCT04468659), primarily aimed to determine the change from baseline of the Preclinical Alzheimer Cognitive Composite 5 score at 216 weeks of treatment, is being conducted to assess the efficacy and safety of lecanemab in preclinical AD patients.

#### Solanezumab

Solanezumab (LY2062430) is a humanized monoclonal antibody that targets the mid-domain of Aβ peptide (Aβ_13-28_) to increase Aβ clearance [47]. Two completed phase III clinical trials, EXPEDITION 1 (NCT00905372) and EXPEDITION 2 (NCT00904683), failed to demonstrate efficacy of solanezumab in retarding cognitive decline and improving functional ability in patients with mild-to-moderate AD [48]. In addition, EXPEDITION EXT (NCT01127633), as an open-label extension study of EXPEDITION 1 and EXPEDITION 2, was terminated as it did not meet the primary endpoint. Meanwhile, two other phase III clinical trials, Expedition 3 (NCT01900665) and ExpeditionPRO (NCT02760602), were terminated due to the failure in improving cognitive decline. In a recent trial (DIAN-TU, NCT01760005) conducted to test the effects of solanezumab on patients with dominantly inherited AD, solanezumab treatment engaged its Aβ targets but showed no improvement and even a little aggravation of cognitive impairment compared to the control group [49]. Although these clinical trials did not show statistically significant benefits for patients with mild to moderate AD, another phase III clinical trial, A4 (NCT02008357), is underway to explore the effects of solanezumab in asymptomatic or very mild patients with amyloid plaques in the brain.

#### Crenezumab

Crenezumab (RG7412) is a humanized IgG1 monoclonal antibody, targeting multiple forms of Aβ, including monomers and aggregates [50]. It has a tenfold higher affinity for oligomers [50]. Two phase III trials, CREAD (NCT02670083) and CREAD2 (NCT03114657), were terminated because a pre-planned interim analysis found unlikeliness to hit the primary endpoint of improving CDR-SB scores. The phase III trial CREAD OLE (NCT03491150) was terminated due to an interim analysis as well. Currently, a phase II clinical trial (NCT01998841) is being conducted to evaluate the efficacy and safety of crenezumab *versus* placebo in preclinical AD patients with presenilin 1 (*PSEN1*) E280A autosomal dominant mutation.

#### Gantenerumab

Gantenerumab (RO4909832) is a human IgG1 monoclonal antibody that binds to aggregated Aβ with high affinity and facilitates Aβ clearance *via* Fc receptor-mediated phagocytosis [51, 52]. In February 2020, gantenerumab was announced to fail to meet the primary endpoint in a phase II trial (DIAN-TU, NCT04623242) on patients with inherited AD. After that, another phase II trial, DIAN-TU-001 (NCT01760005), has been carried out in individuals with mutations associated with early-onset AD. Gantenerumab treatment significantly decreased Aβ plaques, CSF total tau and phospho-tau181 and attenuated the increases of neurofilament light chain, but without benefits for cognitive measurements. Amyloid-related imaging abnormalities edema was observed in 19.2% of the subjects [49]. These results led to the belief that a higher dose of gantenerumab was needed for probable clinical efficacy. Currently, two randomized, double-blind, placebo-controlled, parallel-group phase III trials, GRADUATE 1 (NCT03444870) and GRADUATE 2 (NCT03443973), are ongoing to study the safety and efficacy of gantenerumab in the broader population of people with AD not directly caused by gene mutations. In addition, two open-label, multicenter, rollover phase III trials (NCT04339413 and NCT04374253) are underway to assess the safety and tolerability of long-term administration [53].

## Immunotherapies based on tau protein

Another major hallmark of AD is neurofibrillary tangles, which are made up of abnormally phosphorylated tau (p-tau) protein. Tau is a cytoplasmic protein that can stabilize microtubules through binding to tubulin during its polymerization in normal station [54]. However, hyperphosphorylated tau in AD has a reduced ability to bind microtubules, and eventually causes formation of neurofibrillary tangles and generation of aggregates [54]. Of note, the tau protein appears to be better correlated with the severity of cognitive decline than Aβ in AD patients, indicating that strategies targeting tau should be promising [55–57]. There are three main recognized anti-tau strategies, preventing abnormal tau phosphorylation, inhibiting tau aggregation, and promoting the clearance of tau aggregates. Currently, most anti-tau agents in clinical trials are immunotherapies.

**Fig. 2:**
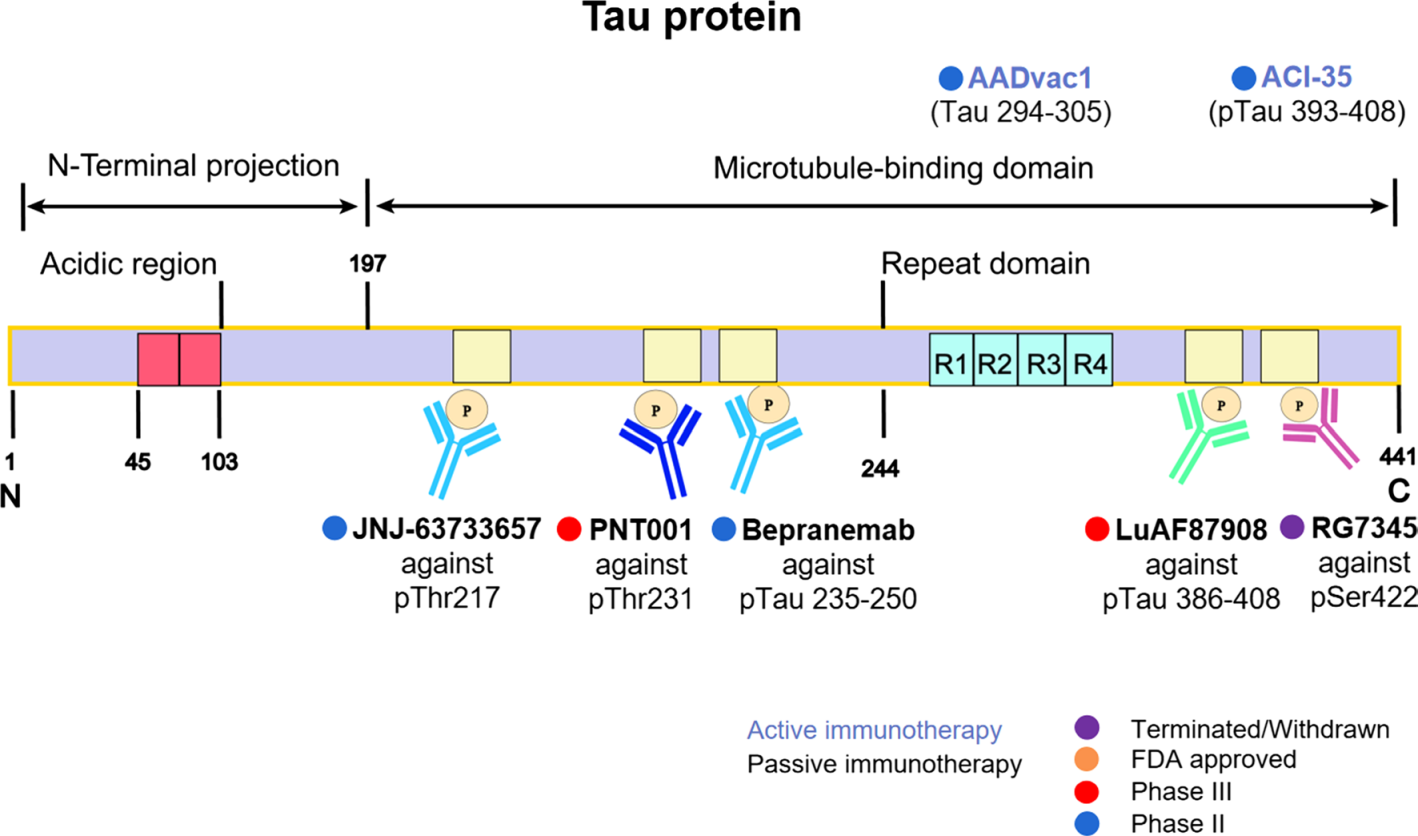
Immunotherapy strategies targeting tau. Tau immunotherapies, including active vaccines and passive antibodies, are shown based on their target region or site

Since the tau immunotherapy was first reported effective in the JNPL3 mice model in 2007 [58], active vaccines like AADvac1 and ACI-35 and passive immunotherapeutic antibodies such as semorinemab, gosuranemab, and BIIB076 have emerged in recent years. They all have significant therapeutic effects in AD animal models [55], and most of them have entered clinical research. The details are shown in Fig. 2 and Table 2.

**Table 2:** Therapeutic strategies targeting tau in AD drug development

Therapeutic strategy	Drug	Mechanism	Sponsor	Study population	Admin	Phase	Results	Clinical Trial Identifier	Start date	Estimated end date
Active immunotherapy	AADvac1	Active vaccine	Axon Neuro- science SE	Mild AD	IV	II	Completed	NCT02579252	2016 Mar	2019 Jun
	ACI-35	Active vaccine	AC Immune SA, Janssen	Early AD	IV	I/II	Recruiting	NCT04445831	2019 Jul	2023 Oct
Passive immunotherapy	RG7345 (RO6926496)	Monoclonal antibody	Discontinued	Healthy	IV	I	Completed	NCT02281786	2015 Jan	2015 Oct
	BIIB076 (NI-105)	Monoclonal antibody	Biogen	Healthy and AD	IV	I	Completed	NCT03056729	2017 Feb	2020 Mar
	Semorinemab (RO7105704)	Monoclonal antibody	Genentech	Mild AD	IV	II	Completed	NCT03289143	2017 Oct	2021 Jan
			Genentech	Moderate AD	IV	II	Active, not recruiting	NCT03828747	2019 Jan	2023 Oct
	Tilavonemab (ABBV-8E12)	Monoclonal antibody	AbbVie	Early AD	IV	II	Completed	NCT02880956	2016 Oct	2021 Jul
			AbbVie	Early AD	IV	II	Active, not recruiting	NCT03712787	2019 Mar	2021 Jul
	Zagotenemab (LY3303560)	Monoclonal antibody	Eli Lilly	Early AD	IV	II	Active, not recruiting	NCT03518073	2018 Apr	2021 Oct
	Gosuranemab (BIIB092)	Monoclonal antibody	Biogen,	Mild AD	IV	II	Terminated	NCT03352557	2018 May	2021 Aug
	PNT001	Monoclonal antibody	Pinteon Therapeutics	Healthy	IV	I	Completed	NCT04096287	2019 Sept	2021 Feb
	Lu AF87908	Monoclonal antibody	H. Lundbeck	Healthy and AD	IV	I	Recruiting	NCT04149860	2019 Sept	2022 Jul
	JNJ-63733657	Monoclonal antibody	Janssen	Early AD	IV	II	Recruiting	NCT04619420	2021 Jan	2025 Mar
	E2814	Monoclonal antibody	Eisai	Mild to moderate AD	IV	I/II	Recruiting	NCT04971733	2021 Jun	2024 Apr
	Bepranemab (UCB0107)	Monoclonal antibody	Hoffmann La Roche, UCB S.A	Mild cognitive impairment or mild AD	IV	II	Recruiting	NCT04867616	2021 Jun	2025 Nov

AD, Alzheimer’s disease; Admin, Route of administration; IV, intravenous

The pharmacological mechanisms of active vaccine involve stimulation of patient’s immune system by administering phosphorylated or non-phosphorylated tau to cause an immune response to produce endogenous antibodies against tau protein [55]. Tau immunogens with mild adjuvants are shown effective in decreasing the pathological tau levels while not inducing severe adverse immune reactions [55]. ACI-35 and AADVac1 remain the only two active vaccines in clinical research for nearly a decade and AADVac1 is the first product entering phase II trial (NCT02579252) [59, 60].

Passive antibodies are designed to recognize different sites of tau protein, which offer a safer option than active vaccines in reducing the risk of immunological adverse effects. In addition, passive immunization also provides greater specificity for targeted epitopes. It has been demonstrated that the passive antibodies can enter neurons to target intracellular tau proteins, which is mediated by receptor or bulk endocytosis [61]. Besides, anti-tau antibodies are also able to curb AD progression by preventing the spread of extracellular tau [61]. Up to date, 11 antibodies have entered clinical trials, and 7 of them are still in clinical test. These antibodies target the microtubule-binding domain (235–250, 386–408), the pThr217, the pThr231, the pSer422, etc. (Fig. 2). However, the development of tau antibody is not as advanced as Aβ, and none of the 7 agents have reached phase III trial. The difficulty of tau antibody research is that the drugs should not only target tau protein in neuron cells but also inhibit tau diffusion outside the cell. This balance is difficult to maintain and adverse reactions with inflammation may occur during treatment [62]. However, considering the important role of tau in AD progression, tau immunotherapy is still worth exploring.

### Active Immunotherapy

#### AADvac1

AADvac1 is the first-generation active immunotherapy vaccine developed by Axon Neuroscience, which targets a 12-amino-acid sequence, KDNIKHVPGGGS, in the microtubule-binding region of tau protein. It is safe with rare adverse events being observed in either immunized or placebo group [63]. Besides, AADvac1 treatment resulted in less brain atrophy and reduced cognitive decline in patients with mild-to-moderate AD in phase I trial (NCT02031198) [64]. And it is exciting that AADvac1 significantly reduced the levels of two CSF biomarkers of AD, p-tau181 and p-tau217 [65]. These positive results support the transition of AADvac1 to phase II trial. Information from clinicaltrials.gov showed that AADvac1 finished a phase II trial on November 14, 2019, but no other details have been reported [63]. Another phase II trial evaluating the safety and tolerability of long-term AADvac1 treatment and the immunogenicity and efficacy of AADvac1 in slowing cognitive decline of AD patients has been completed recently [66]. The results showed that AADvac1 was safe and well tolerated, but it failed to improve cognitive function in a total of 196 patients. There is no doubt that AADvac1 makes a monumental progress in AD active immunotherapy. Nevertheless, larger stratified studies should be conducted to better assess the therapeutic efficacy of AADvac1 in clinical treatment.

#### ACI-35

ACI-35 is the other active immunotherapy vaccine designed by AC Immune to target the pathological conformers of hyperphosphorylated tau. The vaccine contains 16 copies of a synthetic tau recognizing the pathological phosphorylation residues S396 and S404 of tau. ACI-35 is still in a multicenter, double-blind, randomized phase I/II clinical trial to detect the safety and efficacy in mild-to-moderate AD patients in Finland, which is expected to complete before October 31, 2023 (NCT04445831) [67].

### Passive immunotherapy

#### Semorinemab

Semorinemab (RO705705) is a humanized anti-tau monoclonal antibody against extracellular tau with an immunoglobulin G4 isotype backbone which can bind all six human tau isoforms and protect neurons. Its safety profile has been published by AC Immune SA, but no effectiveness signals on AD were observed in clinical trials [68]. Obviously, a further suitably designed trial is required to evaluate its efficiency. A study of semorinemab in patients with moderate AD is ongoing until October 2023 (NCT03828747) [69]. In January 2021, an article published in *Nature Reviews Drug Discovery* reported that semorinemab does not improve AD symptoms and declared the failure of the phase II trial (NCT02754830) [70].

#### BIIB076 and Gosuranemab

BIIB076 (NI-105) and gosuranemab (BIIB092) came from the same company Biogen. BIIB076 is a monoclonal IgG1 targeting the mid-domain of tau. It is still in the early stages of clinical research, and has completed a phase I trial (NCT03056729) [71]. Gosuranemab is a humanized monoclonal antibody against extracellular N-terminal of tau in the interstitial fluid (ISF) and CSF released by neurons. A phase II trial in progressive supranuclear palsy showed that the unbound N-terminal tau in CSF was decreased by 98% in the gosuranemab group and increased by 11% in the placebo group, but the N-terminal tau neutralization does not translate into clinical efficacy (NCT03068468) [72]. Recently, Kim and his colleagues examined the brain tissues of three individuals receiving gosuranemab and found that gosuranemab treatment may be associated with glial responses including accumulation of tau within astrocytic lysosomes [73]. However, the phase II study of gosuranemab in participants with early AD was terminated due to the lack of efficacy in slowing cognitive and functional impairment following the placebo-controlled period readout [74].

#### Tilavonemab

Tilavonemab (ABBV-8E12) is an antibody recognizing the aggregated, extracellular form of pathological tau and binding to the N-terminus of tau. This drug was developed by C2N Diagnostics and AbbVie and has been validated for its safety in a phase I trial (NCT02880956) [75, 76]. However, the phase II trial, evaluating the efficacy and safety of tilavonemab in 453 patients with early AD, did not obtain expected results and now tilavonemab is discontinued in AD treatment (NCT02880956) [76]. In addition, a phase II trial purposed to assess the long-term safety and tolerability of tilavonemab in 364 participants with early AD was finished in September 30, 2021, but its final reports are not available (NCT03712787) [77].

#### Bepranemab

Bepranemab (UCB0107) is a humanized, monoclonal IgG4 antibody from company UCB S.A., targeting the mid-region of tau (amino acids 235–250). The mid-region antibodies seem to be more potential in interfering with the cell-to-cell propagation of pathogenic and aggregated tau than the N-terminal-targeting anti-tau antibodies [78]. Recently, a phase II study to test the efficacy, safety, and tolerability of bepranemab in mild AD patients is under enrollment (NCT04867616) [79]. This trial was estimated to include 450 participants and expected to be finished in November 2025.

#### Zagotenemab

Zagotenemab (LY3303560) is a humanized anti-tau antibody derived from MCI-1, Peter Davies' mouse monoclonal antibody against an early pathological conformation of tau. Until now, zagotenemab has completed two phase I trials on healthy participants and AD patients to examine the safety of repeated doses (NCT02754830, NCT02754830) [80, 81]. A phase II trial finished enrollment of 285 people with at least six months of gradual and progressive memory decline in August 2019 and ran until August 2021. In October 2021, Lilly announced that the trial had missed its primary endpoint, and the development of zagotenemab was terminated [82].

#### JNJ-63733657, E2814, Lu AF87908, PNT001 and RG7345

Several other tau antibodies are currently in the early drug development stage for AD and other tauopathies. JNJ-63733657 is a humanized IgG1 monoclonal antibody designed by Janssen. This antibody can recognize the microtubule-binding region of tau with high affinity for pThr217. JNJ-63733657 treatment has been shown to cause dose-dependent reductions of pTau in the CSF [83]. Now, JNJ-63733657 is in the phase II to evaluate its effect on cognitive decline in AD patients (NCT04619420) [84]. E2814 and Lu AF87908 are humanized, monoclonal IgG1 antibodies from Eisai Co. and Lundbeck, respectively. E2814 recognizes an HVPGG epitope in the microtubule-binding domain near the mid-domain of tau [85], while LuAF87908 targets pSer396 and pSer404. Until now, research on the two antibodies is still in the early stage, with an ongoing phase I/II trial of E2814 to assess its safety and target engagement in mild AD participants (NCT04971733) [86], and a phase I trial to investigate the safety of a single dose of Lu AF87908 in healthy subjects and AD patients under recruitment (NCT04149860) [87]. PNT001 from Pinteon Therapeutics is a monoclonal antibody to the *cis*-isomer of tau phosphorylated at threonine 231 and completed a phase I trial in healthy adults in 2021 while the results have not been published (NCT04096287) [88]. RG7345 (RO6926496) is another antibody against pSer422 of tau, but it has been terminated because of the inflammatory response (NCT02281786) [89].

## Immunotherapy based on microglia

Microglia are thought to play an important role in neuro-immune response and inflammation in the central nervous system. Aβ and phosphorylated tau protein, as damage-associated molecular patterns, are thought to be recognized by receptors such as TLR-4 on the surface of microglia, promoting the release of inflammatory factors in AD brains. The inflammatory factors, in turn, increase the formation of Aβ deposits and neurofibrillary tangles, thus creating a vicious cycle that exacerbates the disease process [90, 91]. A recent PET imaging study in 130 individuals has shown that the interaction between Aβ and activated microglia determines how fast tau spreads across Braak stages, underscoring the intimate connection between microglia and these pathological proteins in the pathological process of AD [92]. In addition, a growing number of genome-wide association studies have demonstrated that many AD risk genes, such as *TREM2*, are highly expressed on microglia, indicating that these molecules can be promising targets for antibodies to modulate microglial function and the neuro-immune system in the brain [93].

### AL002

TREM2 is a key receptor that is selectively expressed by microglia in the brain. Some *TREM2* variants have been identified to increase the risk of late-onset AD [94]. Currently, a TREM2 agonistic antibody binding the extracellular domain of TREM2, which activates the downstream signaling of TREM2 receptor, could cause microglia proliferation and subsequently reduce AD pathology in an AD mouse model [94]. Considering the exciting preclinical results, a few clinical trials based on TREM2 antibody have been initiated. AL002 is a humanized monoclonal IgG1 antibody targeting TREM2, developed in a partnership between Alector and AbbVie. By binding to the microglial receptor TREM2, AL002 activates TREM2 signaling, increases the phosphorylation of TREM2 downstream effector Syk and induces microglia proliferation [95]. Currently, AL002 is in a phase II trial to evaluate its efficacy and safety in 265 participants with early AD. The duration of this study is two years until August 2023 [96].

### Daratumumab

Daratumumab (DARZALEX) is a human antibody targeting CD38 and has been approved by FDA for the treatment of multiple myeloma. Daratumumab can cross the blood–brain barrier and has immunomodulatory activity on nonplasma cells that express CD38 [14]. CD38^+^ CD8^+^ T-cells are significantly increased in the blood of early AD patients and can traffic to the central nervous system to cause toxic effects [97, 98]. A phase II clinical trial is ongoing to explore whether Daratumumab administration has a beneficial effect on patients with mild-to-moderate AD, with an estimated completion date in June 2022 [14].

### Sodium oligomannate

Recently, gut microbiota has gained increasing attention due to its role in AD-associated microglial activation and inflammation [99–102]. Sodium oligomannate, a mixture of acidic linear oligosaccharides derived from the extract of marine brown-algae, gained conditional approval by China’s National Medical Products Administration for the treatment of mild to moderate AD in November 2019 [103] and was included in China's national medical insurance catalog in December 2021. Although sodium oligomannate is not regarded as a traditional immunotherapeutic drug, it reduces microglial activation and neuroinflammation, and consequently slows cognition impairment by regulating gut microbiota and the gut-brain axis [104]. Currently, an international phase III trial for sodium oligomannate in mild to moderate AD patients has been started in North America, Europe, and Asia.

## Challenges and perspectives of immunotherapy for AD

Although the approval of human monoclonal Aβ antibody aducanumab raises the possibility that neurodegeneration in AD may be slowed down or prevented by promoting Aβ clearance, it is still unclear how close we are to the complete treatment of the disease. It cannot be denied that immunotherapy may be the most advanced  disease-modifying strategy for AD treatment, but some issues still need to be noted.

**Fig. 3:**
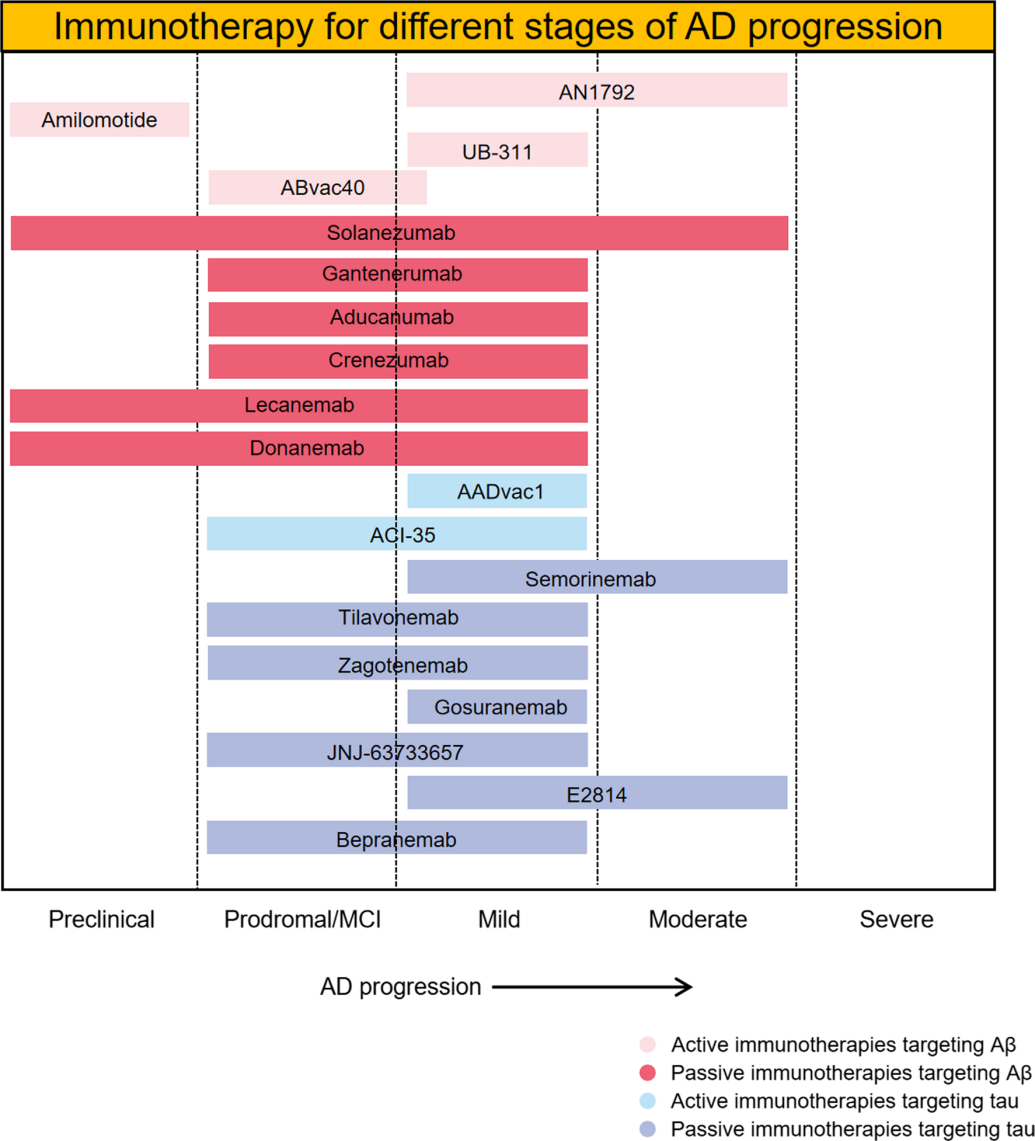
Immunotherapies for AD in ongoing clinical trials. Immunotherapies for different stages of AD progression are shown. Most of the current immunization therapies target the early phase of AD

1. A key step in the development of disease-modifying interventions for AD is to identify appropriate targets. The Aβ and tau hypotheses have been proposed for many years, and other assumed mechanisms such as neuroinflammation, are also associated with these two proteins. However, the relationship between the formation or aggregation of these two proteins and cognition has been debated. Postmortem studies have demonstrated that some patients diagnosed with AD have no Aβ deposits in the brain, while others having Aβ plaques in the brain do not show cognitive impairment. Therefore, it is imperative to more clearly identify Aβ and tau as effective targets for disease-modifying AD treatment, or to consider other critical molecules that drive AD progression. Some uncommon immunotherapy targets, such as TREM2, CD38 and TNF-α, have been tested in clinical trials [14, 95, 96, 105]. With recent advances in single-cell RNA sequencing and spatial transcriptome sequencing, more promising molecules will be found and targeted by immunotherapy.
2. As different forms of Aβ have been observed in the brains of AD patients, it is imperative to identify which form(s) of Aβ should be targeted by immunotherapy to achieve better therapeutic effects. Increasing evidence has reported that soluble Aβ oligomers are more neurotoxic than fibrillary aggregates, and correlate better with AD clinical symptoms [106–108]. The toxic Aβ oligomers have been suggested to be engulfed and processed by microglia to form dense Aβ plaque with relatively low neurotoxicity [109]. Therefore, clearance of Aβ plaque rather than Aβ oligomers might have limited effects on ameliorating the cognitive impairment in AD patients. In addition to Aβ_1-40_ and Aβ_1-42_, pyroglutamate Aβ_3-42_ and Aβ_4-42_ have also been identified as major Aβ isoforms that play significant roles in AD neurodegeneration, with no obvious differences between familial and sporadic AD patients, indicating that these two isoforms can be potential immunotherapeutic targets [110]. Recently, a novel, pseudo β-hairpin conformation of the N-terminal region of pyroglutamate Aβ monomers has been reported, which provides novel insights into both active and passive immunization against AD [111]. Of note, donanemab, a humanized antibody against an N-terminal pyroglutamate Aβ epitope, slows cognitive decline and reduces plaque load in patients with early symptomatic AD in a phase II clinical trial [45]. Taken together, the mechanism of action against different forms, isoforms and epitopes of Aβ might prove a game changer and should be emphasized in AD immunotherapy.
3. Similar to Aβ immunotherapy, various factors may influence the efficacy of tau vaccines or antibodies. Among them, the site of action (extracellular or intracellular) and the epitope of tau may be two most important points. It is of great importance to know which form of tau exists inside the neuronal cells, and which form of tau spreads into other cells to cause the seeding of tau pathology in AD. Anti-tau antibodies can cross into the brain and enter neurons to target intracellular tau proteins, or block the spread of tau pathology by binding extracellular tau [61]. It is reported that the level of CSF tau, which is mainly composed of tau fragments spanning 150–250 amino acids, is increased in AD, indicating that antibodies that target amino acids 150–250 may have benefits in preventing the spread of extracellular tau [61, 112, 113].
4. A growing number of studies has suggested that discrepancies may exist in the pathogenesis and severity of AD patients, and precise diagnosis and individualized treatment of AD patients may be more effective. It is generally regarded that the pathological Aβ occurs earlier than tau in AD brains. Given the different emerging times and roles of Aβ and tau during AD progression, Aβ immunotherapies are more likely to have beneficial effects at the onset or at the early stage of AD with minimal clinical symptoms, such as aducanumab, while the tau immunization strategy would be more effective in slowing cognitive decline in patients with moderate or severe AD. In fact, most of the subjects in clinical trials of both Aβ and tau immunotherapy are patients with early or mild AD (Fig. 3), which underscores the significance of early diagnosis and treatment in AD therapeutic strategy. Nevertheless, the lack of accurate biomarker or cognitive scale has restricted the early diagnosis of AD. There is an imperative need for effective early diagnosis and treatment.
5. Both vaccines and antibodies are administered peripherally, and only a small percentage of drugs can enter the brain. Therefore, methods to improve the delivery efficiency of vaccines or antibodies to across the blood–brain barrier need to be developed to ensure sufficient delivery of drugs into the brain. With respect to this issue, bioengineering and delivery technologies may be helpful. Carriers like biomimetic nanoparticles would effectively improve the delivery efficiency.
6. Strategies should also be carried out to reduce adverse effects in immunotherapy research. For vaccines, it is important to avoid autoimmune T-cell activation. Developing vaccines that target the B cell epitope may be a feasible approach. As for monoclonal antibody, Fc-mediated inflammatory responses need to be reduced, and approaches such as single-chain antibodies and Fc-deglycosylated antibodies are under investigation.
7. Currently, few studies have reported pharmacokinetics (PK) and pharmacodynamics (PD) data in immunization therapy. Relevant indicators should be evaluated in greater detail and precision to better assess the efficacy and safety of immunotherapy.
8. A compound that only targets one aspect of AD may not be enough to produce desired clinical effects for such a complex disease. Therefore, an efficient combination of different immunization therapies to treat AD would have great feasibility. For example, an active vaccine that can simultaneously target pathological Aβ and tau may be more clinically effective than vaccines with a single target, but the problem of increased Th responses and antibody concentrations arising from this kind of vaccine should be solved.

## Conclusions and comments

Although the FDA approval of aducanumab has drawn controversy, it cannot be denied that this human monoclonal Aβ antibody raises the possibility that AD neurodegeneration may be slowed down or prevented by Aβ clearance. Also, it will greatly promote research and development of other AD immunotherapies. Research efforts are being made to overcome challenges in immunotherapy for AD, including target selection, adverse reactions, drug delivery and early diagnosis of AD, in the aim of developing more refined vaccines and antibodies. Resolution of these challenges would pave the way for generation of new immunotherapeutic drugs for AD.

## Data Availability

Not applicable.

## Abbreviations

AD: Alzheimer’s disease
Aβ: Amyloid-β
FDA: Food and Drug Administration
APOE: Apolipoprotein E
TREM2: Triggering receptor expressed on myeloid cells 2
TLR: Toll-like receptor
CDR-SB: Clinical Dementia Rating Scale Sum of Boxes
CSF: Cerebrospinal fluid
PET: Positron emission tomography
ADCOMS: Alzheimer’s Disease Composite Score
ADAS-Cog: Alzheimer’s Disease Assessment Scale-Cognitive Section
